# Eicosapentaenoic acid attenuates dexamethasome-induced apoptosis by inducing adaptive autophagy via GPR120 in murine bone marrow-derived mesenchymal stem cells

**DOI:** 10.1038/cddis.2016.144

**Published:** 2016-05-26

**Authors:** B Gao, Y-H Han, L Wang, Y-J Lin, Z Sun, W-G Lu, Y-Q Hu, J-Q Li, X-S Lin, B-H Liu, Q Jie, L Yang, Z-J Luo

**Affiliations:** 1 Institute of Orthopedic Surgery, Xijing Hospital, Fourth Military Medical University, Xi'an 710032, People's Republic of China; 2 Department of Orthopedic Surgery, First Affiliated Hospital, Chengdu Medical College, Chengdu, People's Republic of China; 3 Health Science Center, Shenzhen University, 3688 Nanhai Ave, Shenzhen 518060, People's Republic of China

## Abstract

Long-term use of glucocorticoids is a widespread clinical problem, which currently has no effective solution other than discontinuing the use. Eicosapentaenoic acid (EPA), an omega-3 long chain polyunsaturated fatty acid (n-3 PUFA), which is largely contained in fish or fish oil, has been reported to promote cell viability and improve bone metabolism. However, little is known about the effects of EPA on dexamethasome (Dex)-induced cell apoptosis. In this study, we showed that EPA-induced autophagy of murine bone marrow-derived mesenchymal stem cells (mBMMSCs). Meanwhile, EPA, but not arachidonic acid (AA), markedly inhibited Dex-induced apoptosis and promoted the viability of mBMMSCs. We also observed that EPA-induced autophagy was modulated by GPR120, but not GPR40. Further experiments showed that the mechanism of EPA-induced autophagy associated with GPR120 modulation involved an increase in the active form of AMP-activated protein kinase and a decrease in the activity of mammalian target of RAPA. The protective effect of EPA on Dex-induced apoptosis via GPR120-meditated induction of adaptive autophagy was supported by *in vivo* experiments. In summary, our findings may have important implications in developing future strategies to use EPA in the prevention and therapy of the side effects induced by long-term Dex-abuse.

Dexamethasone (Dex) is a potent synthetic member of the glucocorticoid (GC) class of steroid drugs that are widely used to regulate various developmental and metabolic processes including bone remodeling.^1^ However, side effects including bone loss, low bone mineral density, and increased fracture risk limit long-term use of GCs. Previous studies have shown that long-term and high dosage of Dex induces apoptosis in bone marrow-derived mesenchymal stem cells (BMMSCs). Furthermore, factors that act by inducing apoptosis of BMMSCs can lead to bone loss and various skeletal metabolic diseases.^2, 3^ Owing to the widespread use of this drug in clinical settings, discovering novel solutions to prevent the apoptotic effect of Dex and to inhibit bone loss will be beneficial to patients suffering from skeletal diseases such as osteoporosis.

Polyunsaturated fatty acids (PUFAs), which were for a long time solely considered as an energy source in our bodies, have been proven to be highly biologically active molecules. The two major families of PUFAs are the omega-3 (n-3) and omega-6 (n-6) PUFAs, whose ratio in the body is of higher importance than the absolute levels of a certain fatty acid.^4^ It has been observed that eicosapentaenoic acid (EPA), a member of the n-3 family PUFAs, increases cell proliferation and exerts anti-apoptotic effects via various mechanisms including modulation of autophagy,^4, 5^ whereas arachidonic acid (AA), a member of the n-6 family PUFAs, exerts opposite effects.^6^ GPR120 and GPR40 are G-protein coupled fatty acid receptors, and numerous studies have shown that EPA and AA are endogenous ligands for these receptors,^7, 8^ which have been implicated in many key processes and exert multiple functions.^9, 10, 11^ Our previous works have shown that activation of GPR120 inhibit Dex-induced apoptosis and determine the bi-potential differentiation potency in a dose-dependent manner.^3, 12^ However, it is not known whether EPA or AA, endogenous ligands of GPR120, protect mBMMSCs from Dex-induced apoptosis.

Autophagy is characterized by the sequestration of bulk cytoplasm and organelles in double- or multi-membrane autophagic vesicles and their subsequent degradation by lysosomes for macromolecular synthesis and ATP generation.^13^ Several studies have demonstrated that autophagy regulates cell proliferation and function of osteoclasts,^14^ osteoblasts,^15^ and osteocytes,^16^ suggesting that autophagy is an essential process for bone homeostasis. In addition, our previous study has shown that autophagy is involved in GC-induced rBMSCs damage.^17^

Although evidence suggests that EPA or AA can protect cells from apoptosis, the role of EPA in the induction of the autophagic pathway in mBMMSCs has not yet been examined. It is not known whether autophagy is induced in Dex-induced apoptosis and, if so, how autophagy contributes to cell apoptosis. In the present study, we investigated the modes and molecular mechanisms of mBMMSCs autophagy that are involved in the anti-apoptotic effect of EPA. To the best of our knowledge, this study provides the first evidence that EPA treatment leads to autophagy via GPR120-mediated AMPK/mTOR signaling and that EPA-induced autophagy protects cells from Dex-induced apoptosis. Overall, our results develop a better understanding of a unique mechanism of the protective action of EPA and GPR120 against side effects induced by long-term Dex use.

## Results

Previous and current studies have shown that high concentrations of Dex, especially 10^−6^ M, caused apoptosis in murine BMMSCs (Supplementary Figure 1A, B).^3^ To further elucidate whether Dex induces autophagy in mBMMSCs, cells were treated with increasing concentrations of Dex. Following a 24-h culture of Dex, LC3 level were detected using western blot and reverse transcription polymerase chain reaction (RT-PCR), respectively. The results showed that 10^−8^ and 10^−7 ^M of Dex slightly upregulated the expression of LC3 levels and 10^−9^ and 10^−7 ^M of Dex increased the protein level of LC3 (LC3-2/LC3-1), but the difference from control levels was not statistically significant. Although 10^−6 ^M of Dex markedly induced apoptosis in mBMMSCs, it did not markedly increase LC3 levels, indicating that Dex could not markedly induce autophagy in mBMMSCs (Supplementary Figure 1C, D). In order to evaluate autophagy flux upon Dex treatment, cells were treated in the presence or absence of 10^−6 ^M Dex and 200 nm Bafilomycin A1 (Baf), which is the lysosomal inhibitor to monitor autophagic flux. The results showed that Baf could markedly induce LC3 accumulation and increased LC3 levels no matter Dex was added or not, which further demonstrated that Dex alone could neither markedly induce autophagy nor block autophagy flux (Supplementary Figure 1E, F).

### EPA, but not AA, induced autophagy and inhibited Dex-induced apoptosis in mBMMSCs *in vitro*

**Figure 1:**
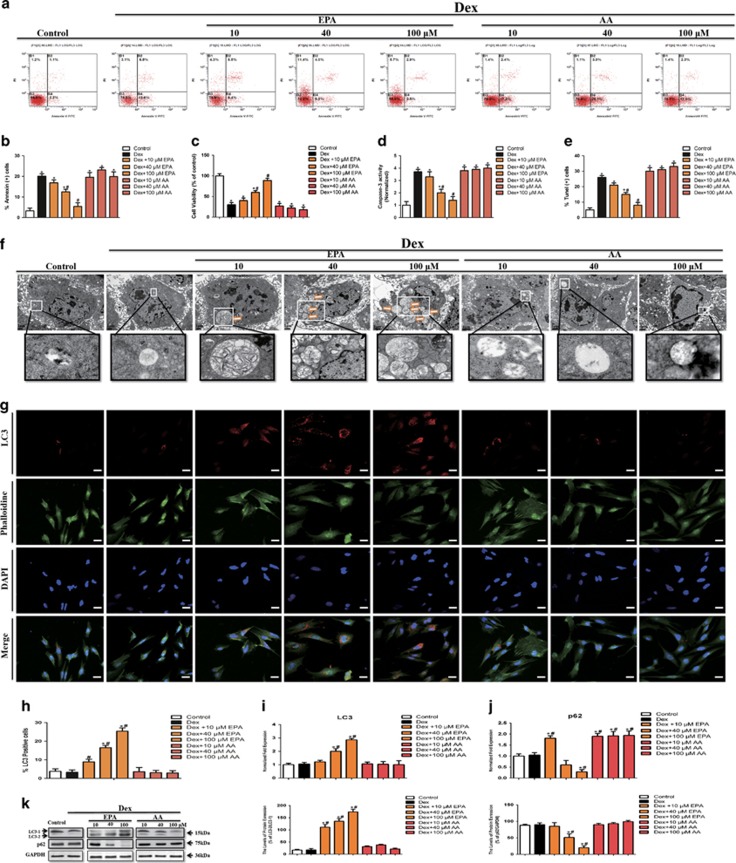
EPA, but not AA, induced autophagy and inhibited Dex-induced apoptosis in mBMMSCs *in vitro*. mBMMSCs were treated with Dex (10^−6 ^M) in the absence or presence of EPA or AA for 24 h. (**a** and **b**) Annexin V/PI double staining was performed to detect apoptotic cells. (**c**) EPA or AA was applied to the mBMMSCs at concentrations of 10, 40, and 100 *μ*m. The cultures were incubated with Dex (10^−6 ^M) for 24 h in the presence or absence of EPA or AA and then cell viability was determined by MTT assay. (**d**) Caspase-3 activity was detected by the caspase-3 assay kit in mBMMSCs that were treated with Dex (10^−6 ^M) in the absence or presence of EPA or AA for 24 h. (**e**) Cell apoptosis was detected by TUNEL assay and number of TUNEL-positive cells as a percentage of the positively stained mBMMSCs. (**f**) Fixed cells processed for thin-section electron microscopy. Arrows indicate the autophagic vacuoles digesting organelles or cytosolic contents (magnification, × 2500). (**g**) Confocal microscopic analysis of mBMMSCs following immunofluorescent staining using an anti-LC3 antibody labeled the cytoskeleton with phalloidine and labeled with the nuclear marker DAPI (magnification, × 200). LC3, red; phalloidine, green; nuclei, blue. (**h**) Number of LC3-positive cells as a percentage of the positively stained mBMMSCs. (**i** and **j**) RT-PCR analysis of LC3 and p62 treated with certain groups. Expression of each target gene was calculated as expression relative to *β*-actin and represented as normalized fold expression. (**k**) Western blot analysis and quantification of LC3 (LC3-2/LC3-1) and p62 (p62/GAPDH) protein treated with certain groups. The data are represented as the mean±S.D. of three independent experiments. *P<0.05 compared with control cells (cells cultured in the same dosages of vehicle without Dex, EPA and AA), ^#^*P*<0.05 compared with 10^−6^ M Dex-treated cells. BMMSCs, bone marrow-derived mesenchymal stem cells; Dex, dexamethasone; PI, propidium iodide; EPA, eicosapentaenoic acid; AA, arachidonic acid, LC3, microtubule-associated protein 1 light chain 3

EPA and AA are derived from essential fatty acids, which can hardly be synthesized *in vivo*. To determine the role of EPA and AA in Dex-induced apoptosis, cells were cultured with 10^−6 ^M Dex in the presence of increasing concentrations of EPA or AA for 24 h. Our results show that treatment with EPA, not AA, decreased the percentage of apoptotic cells induced by Dex (Figures 1a and b) and improved cell viability (Figure 1c). Also, EPA, not AA, induced a dose-dependent inhibition of caspase-3 (Figure 1d). And as shown in Figure 1e, EPA significantly decreased the number of TUNEL-positive cells, whereas AA did not block the effect of Dex. As for autophagy, we observed a dose-dependent accumulation of numerous lamellar structures with cytosolic autophagic vacuoles in mBMMSCs after treatment with 10^−6 ^M Dex in the presence of EPA, but not AA (Figure 1f). Consistent with this observation, immunofluorescence analysis showed that Dex treatment with EPA increased the percentage of LC3-positive cells compared with Dex-treated group and control group (cells cultured with the same dosage of vehicle) (Figures 1g and h). However, we did not observe any marked differences in LC3-positive cells between AA and Dex-treated groups. Because autophagic vesicles also accumulate when autophagic vesicle turnover (autophagosome-lysosome fusion or/and downstream cargo degradation) is inefficient,^18^ we asked whether EPA induces autophagic vacuolisation by impairing autophagic vesicle turnover. We therefore assessed LC3 and p62 levels, which is associated with the completed autophagosomes. As shown in Figures 1i and k, LC3 levels were markedly increased after treatment with EPA compared with the Dex and control groups. In contrast, EPA induced a dose-dependent reduction in p62 level (Figures 1j and k). These findings imply that autophagic vesicle turnover is not impaired by EPA and that EPA-induced autophagic vacuolisation is a consequence of autophagic flux activation. In brief, EPA, not AA, induced autophagic flux activation and inhibited Dex-induced apoptosis of mBMMSCs in a dose-dependent manner.

### EPA inhibited Dex-induced apoptotic cell death via induction of adaptive cell autophagy

**Figure 2:**
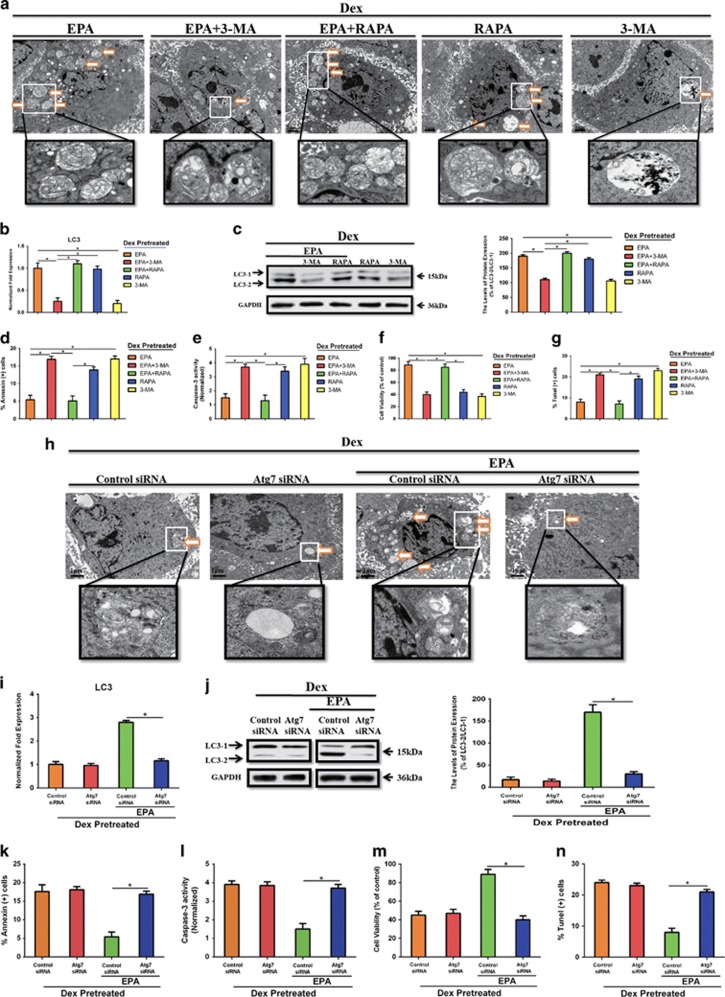
EPA inhibited Dex-induced apoptotic cell death via inducing adaptive cell autophagy. mBMMSCs were cultured with or without 10^−6^ M Dex and 100 *μ*M EPA for 24 h in the presence or absence of 100 nM rapamycin (RAPA) and 5 mM 3-methyladenine (3-MA). (**a**) Fixed cells processed for thin-section electron microscopy. Arrows indicate the autophagic vacuoles digesting organelles or cytosolic contents (magnification, × 2500). (**b**) RT-PCR analysis of LC3 treated with certain groups. (**c**) Western blot analysis and quantification of LC3 (LC3-2/LC3-1) protein treated with certain groups. (**d**) Annexin V/PI double staining was performed to detect apoptotic cells. (**e**) Caspase-3 activity was detected by the caspase-3 assay kit in mBMMSCs that were treated with or without 10^−6^ M Dex and 100 *μ*M EPA for 24 h in the presence or absence of 100 nM rapamycin (RAPA) and 5 mM 3-methyladenine (3-MA). (**f**) The cultures were incubated with or without Dex (10^−6^ M) and 100 *μ*M EPA for 24 h in the presence or absence of 100 nM rapamycin (RAPA) and 5 mM 3-methyladenine (3-MA). Cell viability was determined by MTT assay. (**g**) Cell apoptosis was detected by TUNEL assay and number of TUNEL-positive cells as a percentage of the positively stained mBMMSCs. Expression of each target gene was calculated as a relative expression to *β*-actin and represented as normalized fold expression. (**h**) Atg7 was knocked down by siRNA and cells were cultured with 10^−6^ M Dex with or without 100 *μ*M EPA. Fixed cells processed for thin-section electron microscopy. Arrows indicate the autophagic vacuoles digesting organelles or cytosolic contents (magnification, × 2500). (**i** and **j**) RT-PCR, western blot analysis and quantification of LC3 (LC3-2/LC3-1) treated with certain groups. (**k**) Annexin V/PI double staining was performed to detect apoptotic cells. (**l**) Caspase-3 activity was detected by the caspase-3 assay kit in control or Atg7 knocked down mBMMSCs. (**m**) Cell viability was determined by MTT assay. (**n**) Cell apoptosis was detected by TUNEL assay and number of TUNEL-positive cells as a percentage of the positively stained mBMMSCs. The data are represented as the mean±S.D. of three independent experiments. *P<0.05 compared with each group (**a**–**g**). *P<0.05 compared with control siRNA treated with EPA (**i**–**n**). 3-MA, 3-methyladenine; RAPA, rapamycin

Because EPA triggered autophagy and simultaneously inhibited Dex-induced apoptosis *in vitro*, we aimed to determine whether autophagy affected EPA-induced anti-apoptotic cell death. BMMSCs were cultured with 10^−6 ^M Dex and 100 *μ*M EPA for 24  h in the presence or absence of 100 nM rapamycin (RAPA) and 5 mM 3-methyladenine (3-MA). Moreover, we detected the effect of RAPA and 3-MA alone in the presence of Dex. As shown in Figure 2a, autophagic vacuoles was increased in mBMMSCs after treatment with 10^−6 ^M Dex and 100 *μ*M EPA in the presence of 100 nM RAPA, whereas 5 mM 3-MA inhibited the accumulation of autophagic vacuoles. In total, 5 mM 3-MA also strongly reduced LC3 level induced by EPA, whereas RAPA increased it (Figures 2b and c). Moreover, inhibition of autophagy by 5 mM 3-MA partially blocked the protective effect of EPA in the inhibition of Dex-induced apoptosis and markedly increased the apoptotic cell death, as determined by the increased percentage of apoptotic cells, lower cell viability, increased levels of caspase-3 activity and the increased number of TUNEL-positive cells (Figures 2d–g). Activation of autophagy by RAPA further augmented the protective effect of EPA, but there were no significant differences between these two groups (EPA and EPA+RAPA). Although RAPA alone could induce cell autophagy at large extent, it could not inhibit Dex-induced apoptosis, which indicated that EPA might have some bonus on cell viability and directly protected cells from Dex-induced apoptosis and the type of EPA- or RAPA-induced autophagy might not be the same. To better address this point, we wanted to know whether EPA was able to induce autophagy without Dex and whether the LC3 induction effect of EPA was caused by blocking autophagy flux. Cells were treated with or without 10^−6^ M Dex and 100 *μ*M EPA in the presence or absence of 200 nM Baf. The result showed that EPA alone was not able to induce autophagy without Dex as EPA treatment could not markedly increase the LC3 levels compared with Baf or Baf+EPA-treated group without Dex, and Baf could markedly induce LC3 accumulation no matter Dex was added or not, which indicated that EPA could trigger adaptive autophagy due to Dex-induced apoptotic cell death and this effect was not by blocking autophagy flux (Supplementary Figure 2A, B). To further confirm the involvement of EPA-induced autophagy in the protection from Dex-induced apoptosis, we used Atg7 small interfering RNA (siRNA) to block autophagy process and to avoid the influence from other cellular signaling alterations affected by 3-MA. RT-PCR and western blot showed that Atg7 were markedly knocked down (Supplementary Figure 3A, B). Thin-section electron microscopic analysis, RT-PCR, and western blot showed that knockdown of Atg7 markedly inhibited cell autophagy, and EPA could not increase LC3 level and induce any autophagic vacuoles in Atg7 knocked down mBMMSCs (Figures 2h–j). What's more, Atg7 siRNA significantly attenuated the protective effect of EPA in inhibiting Dex-induced apoptosis (Figures 2k–n). Together, these results strongly indicated that EPA-induced adaptive autophagy was related to apoptotic cell death and could at least partially inhibit Dex-induced apoptosis.

### GPR120, but not GPR40, mediates EPA-induced autophagy and the subsequent anti-apoptotic effect

**Figure 3:**
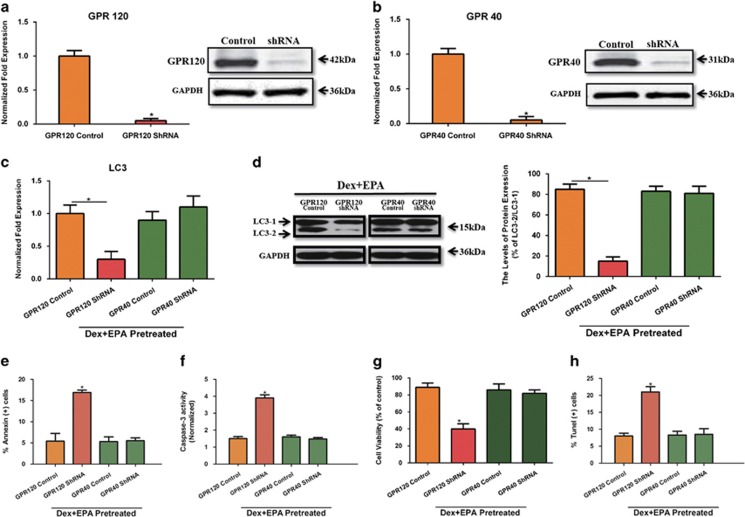
GPR120, but not GPR40, modulated EPA-induced autophagy to inhibit Dex-induced apoptosis. GPR120 and GPR40 were knocked down by shRNA and cells were cultured with 10^−6^ M Dex and 100 *μ*M EPA. (**a** and **b**) RT-PCR and western blot analysis of GPR120 and GPR40. mBMMSCs were invalidated for GPR120 and GPR40 expression or transfected with a non-targeting vector. (**c** and **d**) RT-PCR, western blot analysis, and quantification of LC3 (LC3-2/LC3-1) protein treated with certain groups. (**e**) Annexin V/PI double staining was performed to detect the apoptotic cells. (**f**) Caspase-3 activity was detected by the caspase-3 assay kit in mBMMSCs that were treated with 10^−6^ M Dex and EPA for 24 h. (**g**) Cultures were incubated with Dex (10^−6^ M) and EPA for 24 h and cell viability was determined by MTT assay. (**h**) Cell apoptosis was detected by TUNEL assay and number of TUNEL-positive cells as a percentage of the positively stained mBMMSCs. Expression of each target gene was calculated as a relative expression to *β*-actin and represented as normalized fold expression. The data are represented as the mean±S.D. of three independent experiments. *P<0.05 compared with GPR120 or GPR40 control cells (cells transfected with a non-targeting vector)

GPR120 and GPR40 are G-protein coupled fatty acid receptors, which are located on the membrane of various cell types and have been implicated in many key cellular processes.^7, 19, 20^ Our previous study was the first to investigate the expression and functions of GPR120 and GPR40 in mBMMSCs and showed that GPR120 modulates anti-apoptotic effects in these cells.^3^ In this study, we investigated whether GPR120 and GPR40 affected EPA-induced autophagy and anti-apoptotic cell death in BMMSCs. As shown in Figures 3a and b, GPR40 and GPR120 mRNA and protein level were completely knocked down by using shRNA. We then cultured these cells with 10^−6^ M Dex and 100 *μ*M EPA. The knockdown of GPR120, not GPR40, markedly reduced LC3 level, indicating that GPR120 has an essential role in EPA-induced autophagy (Figures 3c and d). Furthermore, GPR120 shRNA largely impaired the anti-apoptotic effect of EPA compared with GPR120 control group, as shown by the decreased cell viability, increased percentage of apoptotic cells and TUNEL-positive cells and increased levels of caspase-3 activity in GPR120 shRNA-treated cells (Figures 3e–h). However, GPR40 knockdown did not affect EPA-induced autophagy or exerted an anti-apoptotic effect in this culture system.

### EPA induces autophagy through GPR120-medicated AMPK/mTOR signaling

**Figure 4:**
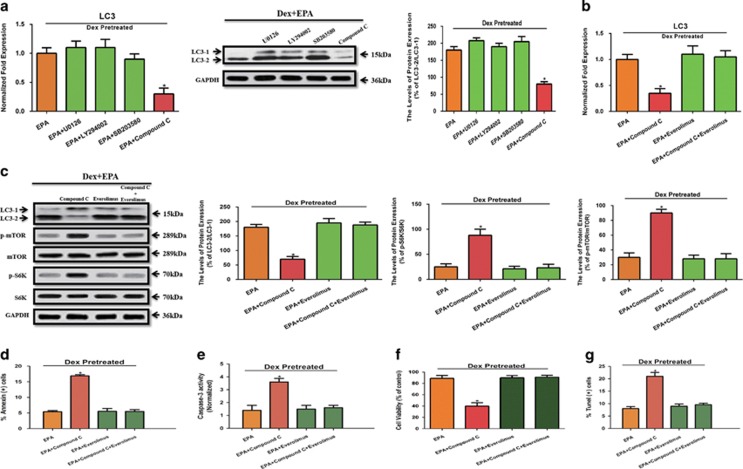
EPA induces autophagy through GPR120-mediated AMPK/mTOR signaling. (**a**) RT-PCR, western blot analysis, and quantification of LC3 (LC3-2/LC3-1) protein. Cells were treated with 10^−6^ M Dex and 100 *μ*M EPA in the presence or absence with the inhibitors of 10 *μ*M Erk1/2 (U0126), 10 *μ*M AKT (LY294002), 10 *μ*M p38 (SB203580), and 10 *μ*M AMPK (Compound C). (**b**) RT-PCR, western blot analysis, and quantification of LC3 (LC3-2/LC3-1) protein. After treatment with 10^−6^ M Dex, cells were cultured with 100 *μ*M EPA in the presence or absence of 10 *μ*M Compound C and 100 nM Everolimus (mTOR inhibitor). (**c**) Western blot analysis and quantification of LC3 (LC3-2/LC3-1), mTOR (p-mTOR/mTOR), and S6K (p-S6K/S6K) protein. (**d**) Annexin V/PI double staining was performed to detect apoptotic cells. (**e**) Caspase-3 activity was detected by the caspase-3 assay kit in mBMMSCs. (**f**) Cultures were incubated with 100 *μ*M EPA in the presence or absence of 10 *μ*M Compound C and 100 nM Everolimus (mTOR inhibitor) for 24 h and cell viability was determined by MTT assay. (**g**) Cell apoptosis was detected by TUNEL assay and number of TUNEL-positive cells as a percentage of the positively stained mBMMSCs. Expression of each target gene was calculated as a relative expression to *β*-actin and represented as normalized fold expression. The data are represented as the mean±S.D. of three independent experiments. *P<0.05 compared with cells cultured with 10^−6^ M Dex and 100 *μ*M EPA

After demonstrating that GPR120-mediated EPA-induced autophagy, we wanted to further elucidate the downstream targets of EPA. mTOR has a pivotal role in the control of autophagy by integrating information from multiple upstream signal transduction pathways and negatively regulating autophagy.^21, 22^ Known downstream targets of GPR120 that may be involved in modulating mTOR signaling mainly belong to four signaling pathways: Ras-Erk1/2,^12^ PI3K-AKT,^23^ p38-MAPK,^24^ and AMPK.^25^ We cultured cells with 10^−6^ M Dex and 100 *μ*M EPA in the presence or absence of inhibitors of Erk1/2 (U0126), AKT (LY294002), p38 (SB203580), and AMPK (Compound C). LC3 mRNA and protein levels were reduced only by treatment with Compound C, whereas the inhibitors of Erk1/2, AKT and p38 could not inhibit EPA-induced autophagy (Figure 4a). Recently, AMPK, a protein complex that responds to changes in cellular AMP/ATP ratio, has been shown to stimulate autophagy via inhibition of mTOR.^26^ We wanted know whether GPR120-mediated AMPK-mTOR signaling activated by EPA was involved in its anti-apoptotic effect. After treatment with 10^−6 ^M Dex, we cultured mBMMSCs with 100 *μ*M EPA in the presence or absence of 10 *μ*M Compound C and 100 nM Everolimus, an mTOR inhibitor. As shown in Figures 4b and c, inhibition of AMPK by Compound C reduced LC3 level, suggesting that mBMMSCs were less likely to undergo autophagy. To better evaluate the direct role of AMPK in the inhibition of mTOR, we detected the total and phosphorylation protein level of mTOR and S6Kinase. The result showed that inhibition of AMPK by Compound C activated mTOR and increased the phosphorylation of mTOR and S6K (Figure 4c). In addition, Compound C decreased the cell viability, increased percentage of apoptotic cells and TUNEL-positive cells and increased caspase-3 activity, suggesting that Compound C increased the number of cells destined for apoptotic cell death compared with EPA treatment (Figures 4d–g). Furthermore, the inhibition of mTOR by Everolimus, even in the presence of Compound C, which markedly upregulated mTOR and induced apoptosis, promoted the anti-apoptotic effect of EPA (Figures 4c–f), indicating that AMPK-mTOR signaling pathway has a pivotal role in EPA-induced autophagy and anti-apoptotic process. Together with our above-mentioned observation that GPR120 regulates EPA-induced autophagy, these correlative data suggest that EPA, but not AA, induces adaptive autophagy to inhibit Dex-induced apoptosis via GPR120 (not GPR40)-mediated AMPK/mTOR signaling.

### EPA protects mBMMSCs from Dex-induced apoptosis and induces autophagy *in vivo*

**Figure 5:**
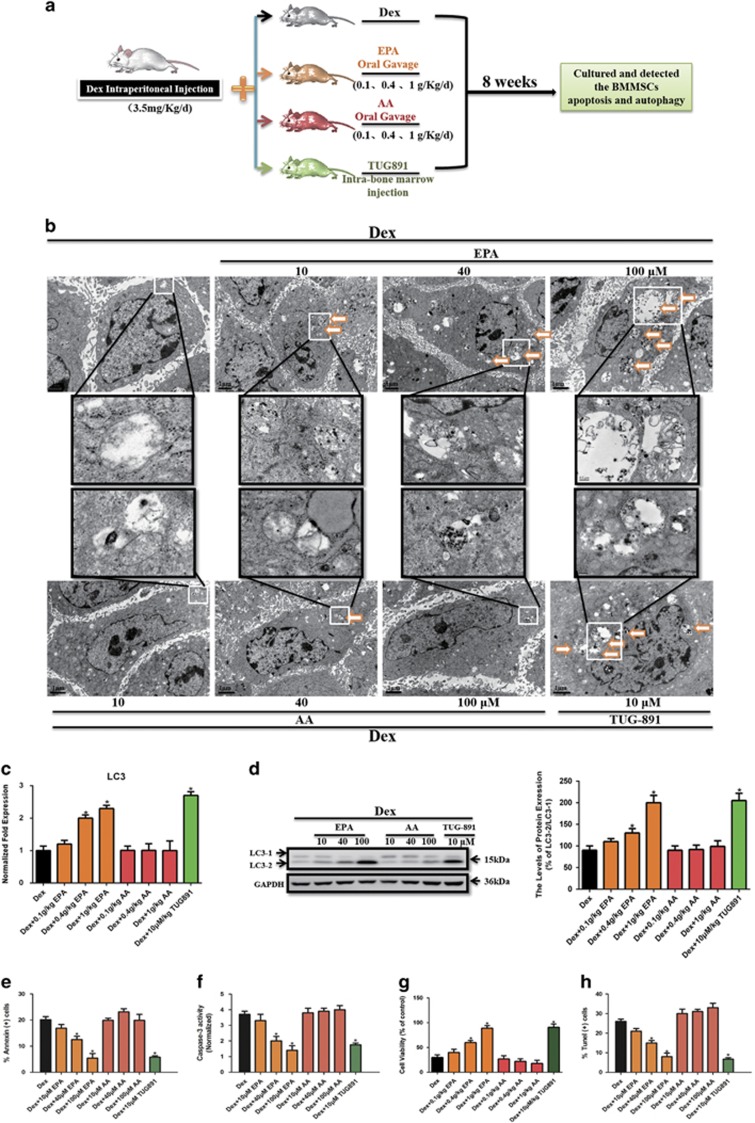
EPA protected mBMMSCs from Dex-induced apoptosis and induced autophagy *in vivo*. Eight-week-old Balb/C mice were treated by intraperitoneal injection of Dex (3.5 mg/Kg/day) in the presence or absence of oral gavage of increasing concentrations of EPA or AA (0.1,0.4,1 g/Kg/day), or by intra-bone marrow injection of TUG-891 (10 *μ*mol/Kg, three times per week, potent and selective agonist of GPR120) for 8 weeks. mBMMSCs of mice from each group were harvested and cultured to investigate the potency of autophagy and apoptosis. (**a**) Diagram of the study and treatment methods. (**b**) Fixed cells processed for thin-section electron microscopy. Arrows indicate the autophagic vacuoles digesting organelles or cytosolic contents (magnification, × 2500). (**c**) RT-PCR, (**d**) western blot analysis and quantification of LC3 (LC3-2/LC3-1) protein. (**e**) Annexin V/PI double staining was performed to detect apoptotic cells. (**f**) Caspase-3 activity was detected by the caspase-3 assay kit in mBMMSCs. (**g**) Cultures were incubated with 10^−6^ M Dex in the presence or absence of increasing concentrations of EPA or AA (10, 40, 100 *μ*M) for 24 h and cell viability was determined by MTT assay. (**h**) Cell apoptosis was detected by TUNEL assay and number of TUNEL-positive cells as a percentage of the positively stained mBMMSCs.. Expression of each target gene was calculated as a relative expression to *β*-actin and represented as normalized fold expression. The data are represented as the mean±S.D. of three independent experiments. *P<0.05 compared with cells cultured with 10^−6^ M Dex

Our *in vitro* experiments demonstrated that EPA, not AA, inhibited Dex-induced apoptosis of mBMMSCs by inducing adaptive autophagy. Based on these findings, we aimed to determine whether similar results would be observed *in vivo*. As shown in Figure 5a, 8-week-old Balb/C mice received an intraperitoneal Dex injection with or without oral gavage of increasing concentrations of EPA, AA, or intra-bone marrow injection of TUG-891, a potent and selective GPR120 agonist^27^ for 8 weeks. We then harvested and cultured mBMMSCs of mice from each group to investigate the potency of autophagy and apoptosis. We observed a dose-dependent accumulation cytosolic autophagic vacuoles in mBMMSCs after treatment with 10^−6 ^M Dex in the presence with EPA, but not AA. In addition, 10 *μ*M TUG-891 had a similar effect as 100 *μ*M EPA of inducing the accumulation of autophagic vacuoles (Figure 5b). Compared with Dex-treated group, EPA markedly upregulated the LC3 level of mBMMSCs in a dose-dependent manner, whereas AA had no effect in this process (Figures 5c and d). Moreover, TUG-891 also increased LC3 level, and the effect was not significant different compared with the group treated with 100 *μ*M EPA. These observations are partially consistent with our *in vitro* data, which showed that EPA-induced autophagy via GPR120 (Figures 5c and d). EPA treatment also markedly inhibited Dex-induced apoptosis *in vivo* in a dose-dependent manner. However, AA had no anti-apoptotic effect and even increased the apoptotic markers (Figures 5e–h). The anti-apoptotic effect induced by TUG-891 was not markedly different from that induced by treatment with 100 *μ*M EPA, which further supports our *in vitro* data that EPA inhibits Dex-induced apoptosis by inducing adaptive autophagy via GPR120-mediated signaling pathways (Figures 5e–h). Taken together, our results demonstrate the role of EPA, but not AA, in inducing adaptive autophagy to protect cells from Dex-induced apoptosis via GPR120-mediated AMPK/mTOR signaling.

## Discussion

GCs have been widely used as potent immunosuppressive drugs for treatment of a variety of inflammatory diseases, including rheumatoid arthritis and inflammatory bowel disease, as well as in transplantation medicine. However, long-term treatment with high doses of GCs causes rapid and sustained bone loss and fragility fractures.^2^ BMMSCs are essential in the maintenance of the dynamic homeostasis of bone tissue, with studies demonstrating that deficiencies in BMMSCs proliferation and osteoblastic differentiation are associated with reduced bone mass.^28, 29^ Our previous work has shown that Dex treatment induced apoptosis and inhibited proliferation of mBMMSCs in a dose-dependent manner. In addition, we have shown that Dex, at doses ranging from 10^−8^ to 10^−6^ M, induces autophagy in rBMMSCs.^17^ Based on these findings, the aim of the current study was to further investigate the role of these two processes in determining cell fate. In this study, we did not observe a marked difference in LC3 between different doses of Dex treatment for 24 h. Although 10^−9^, 10^−7^, and 10^−6^M of Dex increased LC3 levels, the change was modest and not significantly different, indicating that 24 h Dex treatment does not induce autophagy in mBMMSCs. It is noteworthy that in our previous study 10^−7^ and 10^−6^M of Dex-induced autophagy in rBMMSCs. This discrepancy in our findings can be due to different time of cultivation (24 h or 48 h) and species (mice or rat) we use. Indeed, as a process of consuming cellular components and generating energy, autophagy engages in a complex interconnection with apoptosis according to the nature of stimulus and cell type. Autophagy can suppress apoptosis by eliminating damaged organelles in response to cellular stress induced by cancer therapy, or it can sensitize cells to apoptosis in an ATP-dependent process. In this study, we showed that the increased autophagy and decreased apoptosis occurred simultaneously to inhibit Dex-induced cell death in EPA-treated mBMMSCs. In contrast, AA did not only fail to inhibit, but even promoted the Dex-induced apoptosis to some extent. In addition, morphological features of increased autophagy and decreased apoptosis were observed in EPA- but not AA-treated mBMMSCs, further confirming the suggestion that only EPA (not AA) induced autophagy and inhibited Dex-induced apoptosis (Figures 1f and g). This finding further prompted us to closely investigate the connection between these two cellular processes. Any disruption in the process of autophagy, such as autophagosome accumulation and lysosome dysfunction, causes maladaptive autophagy and leads to cell death. In our study, EPA might have some bonus on cell viability and directly protected cells from Dex-induced apoptosis and the type of EPA- or RAPA-induced autophagy might not be the same. Depending on this, we used lysosomal inhibitor Baf and silenced Atg7 by siRNA and we further clarified that EPA could trigger adaptive autophagy owing to Dex-induced apoptotic cell death and this effect was not by blocking autophagy flux. Further studies are needed to determine whether this mechanism is involved in EPA-induced autophagy in apoptosis.

In our previous work, we demonstrated that GPR120 modulates the anti-apoptotic effect of Dex, determines the differentiation potency in a dose-dependent manner and recovers bone metabolism from estrogen-deficiency-induced bone loss.^3, 12^ However, it is not clear whether GPR120 modulates autophagy in mBMMSCs. Therefore, we aimed to determine whether GPR120 modulates the EPA-induced autophagy while inhibiting apoptosis and clarify the mechanism. mTOR has a pivotal role in the control of autophagy and integrates inputs from multiple upstream signal transduction pathways and negatively regulates autophagy. We have demonstrated that Ginsenoside-Rb2 inhibits Dex-induced apoptosis via GPR120-mediated Ras-Erk1/2 cascade and that dose-dependent activation of GPR120 leads to the activation of p38.^3, 12^ Furthermore, studies have shown that GPR120 modulates PI3K-AKT and AMPK in certain cell lines.^23, 25^ Therefore, the known downstream targets of GPR120 that may be involved in modulating mTOR signaling belong to four signaling pathways, namely Ras-Erk1/2, PI3K-AKT, p38-MAPK, and AMPK. We cultured mBMMSCs with the inhibitors of the above signaling pathways in the presence of EPA. Only compound C, an AMPK inhibitor, had the ability to markedly decrease LC3 level (Figures 4a and b), activate mTOR and increase the phosphorylation of S6K (Figure 4c), which clearly demonstrated that AMPK/mTOR signaling pathway has a pivotal role in EPA-induced autophagy via GPR120. It is noteworthy that inhibition of autophagy with AMPK and mTOR inhibitors and GPR120 knockdown using shRNA failed to completely block autophagy treated with EPA, suggesting that EPA-induced autophagy is not exclusively dependent on AMPK-mTOR-mediated activation and that other signaling pathways may participate in the anti-apoptotic process caused by EPA treatment. Further studies are needed to investigate the alternative mechanisms of EPA-induced autophagy. Interestingly, Kyu Lim *et al.* indicated that DHA-induced autophagy was necessary for apoptotic cell death of cancer cells, which seems to contradict our results.^30^ These discrepancies may be due to several factors. First, whereas we used mBMMSCs, Lim *et al.* used SiHa cells. The signaling pathways involved in autophagy or apoptosis-induced cancer cell death are not identical in all cell types. Second, GPR120-mediated cellular changes may be different from p53-mediated alterations. Third, induction of autophagy may differ based on the cell culture system and environment, thus EPA or DHA-induced autophagy processes may be different and exert different effects on modulation of apoptosis. Therefore, different results obtained by Kyu Lim *et al.* and our study suggest that potential therapeutic strategies targeting autophagy may vary depending on the cell types involved and further investigation is necessary to elucidate the complex connection between autophagy and apoptosis.

The last question we addressed is whether the intake of EPA or AA affects the detrimental effects of Dex *in vivo*. GC-induced osteoporosis is a widespread clinical complication of GC therapy. This irreversible damage to bone-forming and -resorbing cells is essential in the pathogenesis of osteoporosis. Previous work has demonstrated that GC administration impaired the proliferation and induced apoptosis of rBMMSCs.^17^ As we know, skeletal growth relies on both biosynthetic and catabolic processes, and autophagy is a catabolic process that has a fundamental part in tissue homeostasis. In this study, consistent with our *in vitro* data, EPA increased LC3 level and protected cells from Dex-induced apoptosis in a dose-dependent manner, whereas AA had no such effect and even promoted the apoptotic cell death induced by Dex (Figure 5). It is worth noting that TUG-891 also blocked Dex-induced apoptosis and induced LC3 level and the effect was similar to that elicited by 100 *μ*M of EPA, which further confirms that GPR120 has a pivotal role in EPA-induced autophagy and Dex-induced apoptosis in mBMMSCs both *in vivo* and *in vitro*.

**Figure 6:**
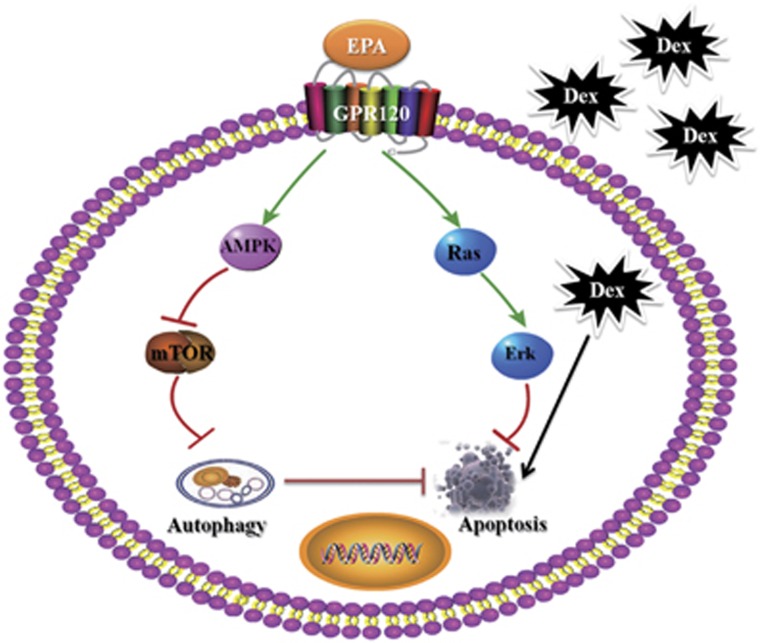
EPA, but not AA, therapy induces adaptive autophagy in Dex-induced apoptotic mBMMSCs and maintains the proliferative ability via GPR120-mediated AMPK-mTOR signaling pathway. Under the circumstance of Dex-induced apoptotic cell death, the downstream targets of GPR120 are highly involved in anti-apoptosis and autophagy-induction process when GPR120 is activated by EPA,. In detail, GPR120 activates Ras-Erk1/2 cascade to inhibit Dex-induced apoptosis and modulates AMPK/mTOR to induce cell autophagy. However, EPA treatment alone without Dex is unable to induce much autophagy, which indicates the effect of EPA in inducing adaptive autophagy when cells are suffering Dex-induced apoptotic cell death

In summary, to the best of our knowledge, our study is the first to show that EPA, not AA, therapy induces adaptive autophagy in Dex-induced apoptosis and maintains the proliferative ability via GPR120-mediated AMPK-mTOR signaling pathway (Figure 6). Collectively, this study highlights the significance of EPA-induced autophagy in bone homeostasis and Dex-induced apoptosis, and this finding may have important implications in developing future strategies to use EPA in the prevention and therapy of the side effects of long-term GC therapy.

## Materials and Methods

### Materials

The a-Modified minimal essential medium was purchased from Thermo Scientific (Waltham, MA, USA). Polystyrene culture dishes were obtained from Costar (Corning, NY, USA), and fetal bovine serum was purchased from Gibco Life Technologies (Waltham, MA, USA). Anti-GPR120 (sc48203) and anti-GPR40 (sc32905) were purchased from Santa Cruz (Dallas, TX, USA). Anti-p62 (ab56416), anti-mTOR (ab32028), anti-p-mTOR (ab109268), anti-S6K1 (ab32529), anti-p-S6k1 (ab5231), anti-Atg7 (ab133528) and anti-LC3B (ab51520) were purchased from Abcam (Cambridge, MA, USA).TUG-891 (4-[(4-Fluoro-4'-methyl[1,1'-biphenyl]-2-yl)methoxy]-benzenepropanoic acid) (4601-50) and U0126 (1144/25) were purchased from R&D Systems (Minneapolis, MN, USA). LY294002 (L9908), SB203580 (S8307) and BafA1 (B1793) were purchased from Sigma Chemical (St. Louis, MO, USA). Penicillin–streptomycin was purchased from Solarbio (Beijing, China). The total RNA extraction kit was purchased from OMEGA (Norcross, GA, USA). The PrimeScript RT reagent kit and SYBR Premix Ex Taq were purchased from TaKaRa (Dalian, China). Other reagents were of the highest commercial grade and purchased from Sigma Chemical.

### Cell treatment and culture

BMMSCs were isolated from Balb/c mice as previously described,^31^ and cells were characterized using mesenchymal stem cell minimal criteria.^32^ Homogenous BMMSCs were obtained through three generations. GC dexamethasone (Dex; Sigma-Aldrich, St. Louis, MO, USA), EPA (Cayman Chemical, Ann Arbor, MI, USA, 90110) and AA (Cayman Chemical, 10007268) dissolved in absolute ethanol, RAPA (Tocris, Bristol, UK, 1292) dissolved in DMSO, and 3-MA (Sigma, St. Louis, MO, USA, M9281) dissolved in PBS were added to the medium to final concentrations as described in each experiment. Cells grown to 60% confluency were switched to serum-free medium and the culture was allowed to expand for 24-h before giving any treatment. For Dex plus EPA or AA treatment, cells were incubated with certain dosages of Dex and EPA or AA for 24-h. For EPA plus RAPA or 3-MA treatment, cells were incubated with certain dosages of Dex and EPA in the presence or absence of 100 nM RAPA and 5 mM 3-MA for 24-h. For Dex plus Baf or EPA treatment, cells were treated in the presence or absence of 100 *μ*M EPA and 200 nM Bafilomycin A1 (Baf) with or without 10^−6^ M Dex.

### Detection of autophagosomes by transmission electron microscopy

Cells were detached from the plates using 0.25% trypsin trypsin-EDTA (Gibco Life Technologies) and fixed with 2% paraformaldehyde/2% glutaraldehyde (Sigma-Aldrich) in 0.2 M sodium cacodylate buffer (pH 7.4; Sigma-Aldrich). Cell pellets were post-fixed with 1% (v/v) osmic acid (Sigma-Aldrich) in sodium cacodylate buffer and were stained with 1% uranyl acetate (Amresco, Solon, OH, USA). Following dehydration, the pellets were embedded in Durcupan (Sigma-Aldrich). Ultrathin sections (50 nm) were prepared using an Ultrotome Ultracut S (Leica Microsystems, Wetzlar, Germany) and images were captured with a JEM-1230 transmission electron microscope (JEOL, Ltd., Tokyo, Japan).

### MTT assay

Cell viability was determined by 3-(4,5-dimethylthiazol-2-yl)-2,5-diphenyltetrazolium bromide (MTT) assay. After treatment with Dexamethason in the presence or absence with EPA or AA for 24-h, cultures were washed with PBS. MTT (0.5 mg/ml) was then added to each well and the mixture was incubated for 4 h at 37 °C. Culture medium was then replaced with an equal volume of DMSO to dissolve formazan crystals. After the mixture was shaken at room temperature for 30 min, absorbance of each well was determined at 550 nm.

### TUNEL assay

Apoptosis in BMMSCs were detected using the DeadEnd Fluorometric TUNEL System (Promega, Madison, WI, USA), which end-labels the DNA from the fragmented apoptotic cells using a modified TUNEL staining according to the manufacturer's instructions. In brief, samples were fixed with 4% methanol-free formaldehyde solution, rinsed with PBS twice, then end-labeled the fragmented DNA of the apoptotic cells using the fluorescein-12-dUTP and covered slides in DAPI to stain nuclei. Samples were immediately analyzed under a fluorescence microscope using a standard fluorescein filter set (Olympus, Tokyo, Japan) to view the green fluorescence of apoptotic cells at 520 nm and blue DAPI-stained nuclei at 460 nm.

### Detection of Caspase-3 activity

Caspase-3 activity was measured spectrophotometrically via the detection of pNA cleavage by caspase-3-specific substrates. These experiments were completed using a Caspase-3 Assay Kit (Beyotime, Shanghai, China). After the cell lysates were incubated with Ac-DEVD-pNA for 2 h at 37 °C, the samples were read at 405 nm.

### Annexin V/PI double staining

The number of apoptotic cells was determined by Annexin V/propidium iodide (PI) double staining. Cells were exposed to 1 *μ*M Dex in the presence or absence of EPA or AA for 24- h and then incubated with FITC-conjugated Annexin V in binding buffer (0.01M HEPES, 0.14M NaCl, and 2.5 mM CaCl2, pH 7.4) for 30 min at 37 °C in the dark. After incubation, the cells were washed and re-suspended in 200 ml PBS with 1% FCS and additionally incubated with 10 ml of 1 mg/ml PI solution. The Annexin V-positive cells were detected using a FACSCalibur flow cytometer (BD Biosciences, San Jose, USA), and the results were analyzed using CellQuest software (BD Biosciences). Annexin V-FITC conjugates were detected with the FL1 channel of the FACSCalibur machine. PI was read on the FL2 channel.

### Immunostaining

Cells were fixed in 4% paraformaldehyde for 15 min, permeabilized with methanol for 10 min, and incubated with primary antibodies anti-LC3B (1:100), phalloidine (1:200) antibodies overnight. On the following day, cells were incubated with a DyLight 594 and 488 secondary antibody (Abcam) for 1 h. After staining nuclei with DAPI (0.5 *μ*g/ml) for 5 min, the cells were analyzed under a FV1000 model confocal microscope (Olympus).

### Western blot analysis

Cells were lysed in lysis buffer (50 mM Tris–HCl pH 7.4, containing 150 mM NaCl, 1% Nonidet P-40, 0.1% SDS, 10 mg/ml leupeptin, 10 mg/ml pepstatin A, and 10 mg/ml aprotinin) on ice for 30 min. For western analysis, 10 *μ*g of total protein was resolved by 10% SDS-PAGE, and proteins were transferred onto a PVDF membrane. Anti-GPR120 (1:1000), anti-GPR40 (1:1000), anti-p62 (1:2000), anti-mTOR (1:5000), anti-p-mTOR (1:5000), anti-S6K1 (1:5000), anti-pS6k1 (1:2000), anti-Atg7 (1:3000), and anti-LC3B (1:1000) antibodies were used for immunoblotting. GAPDH was used as a loading control. The horseradish peroxidase-conjugated secondary antibody was used at a 1:5000 dilution. Images were analyzed by Scion Image software.

### Reverse transcriptase polymerase chain reaction

Total RNA was purified from cells using Trizol (Invitrogen, Waltham, MA, USA). RT-PCR was performed, and results were analyzed as previously described.^33^ All RT-PCR experiments were performed in triplicate, and the primers sequences were as follows: *LC3* (Fwd) ATGCCGTCGGAGAAGACCTT, (Rvs) TTACACTGACAATTTCATCCCG, *GPR120* (Fwd) CCAGTGTTGCTGGAGAAATC, (Rvs) TGATGCCTTGGTGATCTGTA; *GPR40* (Fwd) AATGCTCCAATGTGGCTAGTTTC, (Rvs) GCCAGTGACCAGTGGGTTGA; Atg7 (Fwd) TCTGGGAAGCCATAAAGTCAGG, (Rvs) GCGAAGGTCAGGAGCAGAA; *β-actin* (Fwd) CTGGCACCACACCTTCTACA, (Rvs) GGTACGACCAGAGGCATACA.

### Transfection of lentiviral vectors with shRNA for GPR120 and GPR40

The pGMLV-GFP-vshRNA-GPR120 was constructed (pGMLV, a shRNAi Vector, Shanghai Genomeditech Co. Ltd, Shanghai, China). In the present study, we constructed 3 vshRNA-GPR120 lentiviral vectors (pGMLV-GFP-shRNA-GPR120) and 3 vshRNA-GPR40 lentiviral vectors (pGMLV-GFP-shRNA-GPR40) to silence the expression of GPR120 and GPR40 in murine BMMSCs (BMMSCs-vshRNA-GPR120/40). Three shRNA-targeting sequences for GPR120 were as follows: #1, 5′-GCACCCACTTCCCTTTCTTCT-3′ #2, 5′-GCTCTTCTACGTGATGACAAT-3′ and #3, 5′-GGACCAGGAAATTCCGATTTG-3′. And three shRNA-targeting sequences for GPR40 were as follows: #1, 5′-CCCTGCCCGACTCAGTTTC-3′ #2, 5′-GGCAGCCCACATAGCAGAA-3; and #3, 5′-CCGGGCCCGTCTCAGTTTCTCCATTCTCGAGAATGGAGAAACTGAGACGGGCTTTTT-3′. Stably transfectantclones were characterized by RT-PCR, western blot, and immunoblotting.

### Small interfering RNA transfection

siRNA oligonucleotides specific for Atg7 and the siRNA control were purchased from Santa Cruz Biotechnology (Santa Cruz, CA, USA). Cells were seeded into a 60-mm dish which was then left for 24 h. A 2 ml aliquot of siRNA solution (10 mM) and 5 ml of Lipofectamine 2000 (Invitrogen) were each mixed with 100 ml of serum-free RPMI 1640 medium. They were incubated for 20 min at room temperature after combining the two mixtures, and this was then added to the cells that had been seeded on the dish. After 24 h, harvested cells were subjected to western blot analysis. Cells were also processed for cell viability and apoptosis analysis.

### Animals and experimental procedures

A total of 40 healthy female Balb/c mice (4 months old; average weight, 22.1±1.4 g) obtained from the Laboratory Animal Centre of Fourth Military Medical University (Xi'an, China) were randomly divided into eight groups with five mice in each group. Randomization was performed using a random number table. The groups were divided as follows: (1) Dex, 3.5 mg/kg/d distilled water, Intraperitoneal Injection; (2–4) Dex+EPA (0.1, 0.4, 1 g/kg/day), intraperitoneal injection of Dex and oral gavage of EPA; 5-7) Dex+AA (0.1, 0.4, 1 g/kg/day), intraperitoneal injection of Dex and oral gavage of AA; (8) Dex+TUG-891, intraperitoneal injection of Dex and intra-bone marrow injection of TUG-891 (10 *μ*M/kg). Dex, EPA and AA treatments were administered daily and TUG-891 treatment was administered three times per week, for 8 weeks prior to sacrifice by cervical vertebra luxation. All experimental procedures were approved by the Institutional Ethics Review Board of Xijing Hospital (permission code 20110405-5).

### Statistical analysis

Data were expressed as means±S.D. of multiple repeats of the same experiment (*n*=5). The data for these measurements were analyzed by one-way analysis of variance with subsequent *post hoc* multiple comparisons by Dunnett's test. Statistically significant values were defined as *P*<0.05.

## Abbreviations

EPA: eicosapentaenoic acid
AA: arachidonic acid
Dex: dexamethasome
BMMSCs: bone marrow-derived mesenchymal stem cells
PUFA: polyunsaturated fatty acid
LC3: microtubule-associated protein light chain 3
GPR120: G-protein coupled fatty acid receptor 120
