# Supplementary figures and images for: Eicosapentaenoic acid attenuates dexamethasome-induced apoptosis by inducing adaptive autophagy via GPR120 in murine bone marrow-derived mesenchymal stem cells
Source: Cell Death Dis. 2016 May 26;7(5):e2235–. doi: 10.1038/cddis.2016.144 (PMC4917672; doi:10.1038/cddis.2016.144)

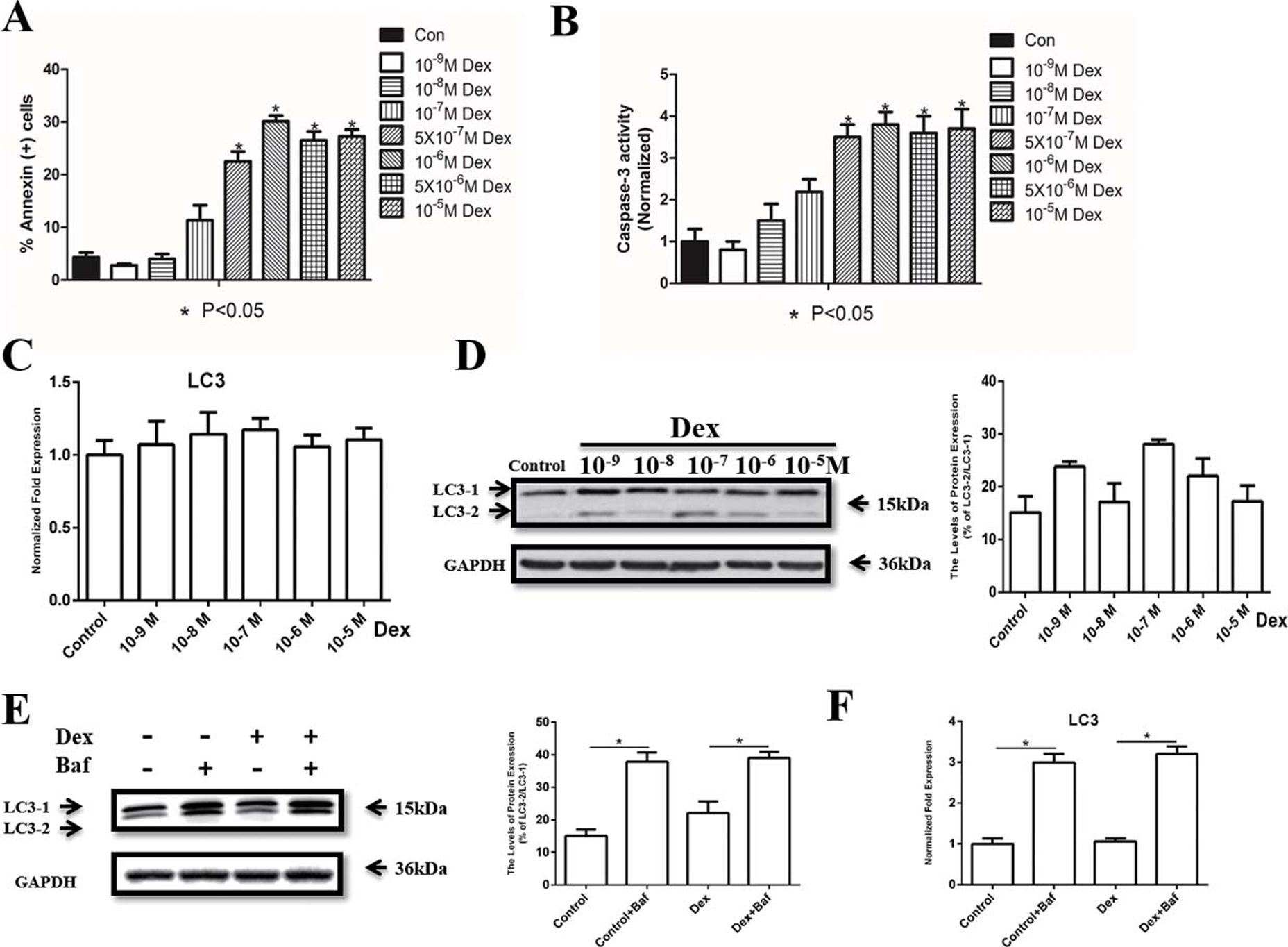

Supplement: Supplementary Figure 1 [file cddis2016144x1.tif]

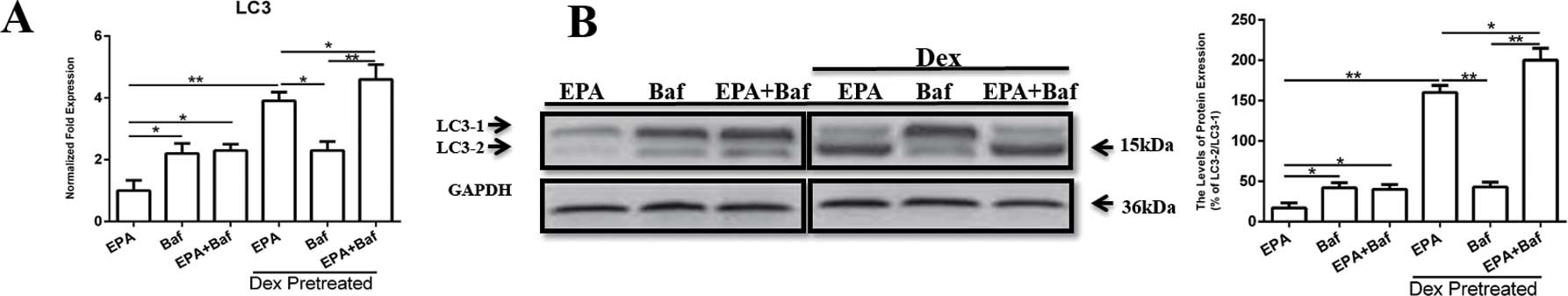

Supplement: Supplementary Figure 2 [file cddis2016144x2.tif]

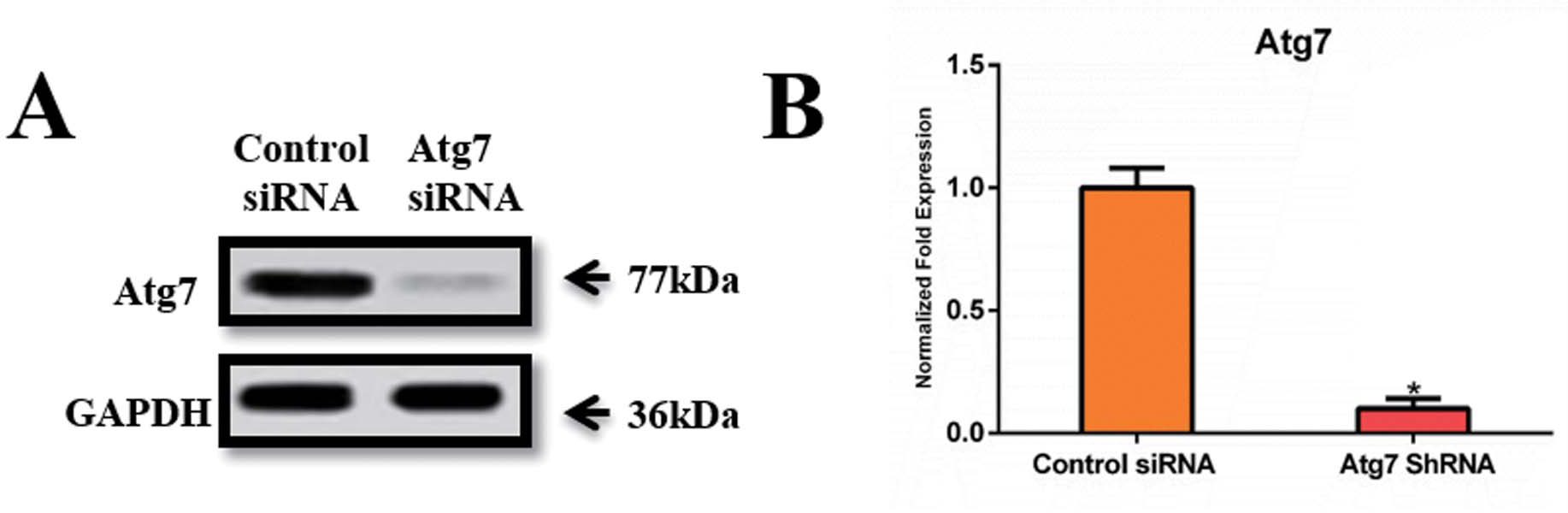

Supplement: Supplementary Figure 3 [file cddis2016144x3.tif]
