# Supplementary material for: Eicosapentaenoic acid attenuates dexamethasome-induced apoptosis by inducing adaptive autophagy via GPR120 in murine bone marrow-derived mesenchymal stem cells
Source: Cell Death Dis. 2016 May 26;7(5):e2235–. doi: 10.1038/cddis.2016.144 (PMC4917672; doi:10.1038/cddis.2016.144)
Supplement: Supplementary Figure Legends [file cddis2016144x4.docx]

**Supplementary Figure 1**

**Dex induced apoptotic cell death in a dose-dependent manner, but had no markedly effects in the induction of autophagy in mBMMSCs.** mBMMSCs were cultured with increasing concentrations of Dex (10^-9^,10^-8^,10^-7^,10^-6^ and 10^-5^ M). (A) Annexin V/PI double staining was performed to detect apoptotic cells. (B) caspase-3 activity was detected by the caspase-3 assay kit (C,D) RT-PCR, western blot analysis and quantification of LC3 (LC3-2/LC3-1) protein. (E,F) mBMMSCs were cultured with or without 10^-6^ M Dex and 200nM Bafilomycin A1 (Baf). Western blot analysis and quantification of LC3 (LC3-2/LC3-1) protein and RT-PCR analysis of LC3 mRNA. Expression of each target gene was calculated as a relative expression to β-actin and represented as normalised fold expression. The data are represented as the mean±SD of 3 independent experiments. *P<0.05 compared with control cells (cells treated with same dosage of vehicle) or certain group showed in the graph.

**Supplementary Figure 2**

**EPA treatment alone without Dex is unable to induce cell autophagy.** mBMMSCs were cultured with or without 10^-6^ M Dex in the presence or absence with 100μM EPA and 200nM Bafilomycin A1 (Baf)**.** (A) RT-PCR analysis of LC3. (B) Western blot analysis and quantification of LC3 (LC3-2/LC3-1) protein. Expression of each target gene was calculated as a relative expression to β-actin and represented as normalised fold expression. The data are represented as the mean±SD of 3 independent experiments. *P<0.05 compared with certain group showed in the graph.

**Supplementary Figure 3**

**Atg7 was knocked-down by siRNA.** (A) Western blot analysis of Atg7. (B) RT-PCR analysis of Atg7. Expression of each target gene was calculated as a relative expression to β-actin and represented as normalised fold expression. The data are represented as the mean±SD of 3 independent experiments. *P<0.05 compared with control siRNA.
